# Solvent‐Guided Chiral Assemblies and Chemical Doping of OEG‐Functionalized Conjugated Polymers

**DOI:** 10.1002/adma.74130

**Published:** 2026-07-18

**Authors:** Sanghyun Jeon, Justin Scott Neu, Nahyun Ahn, Ayse Senlik, Yen‐Chi Chen, Pravini S. Fernando, Priyotosh Bairagya, Chetan Kumar Prasad, Janice Mihyun Baek, Wei You, Ying Diao

**Affiliations:** ^1^ Department Materials Science and Engineering University of Illinois at Urbana‐Champaign Urbana USA; ^2^ Department of Chemistry University of North Carolina at Chapel Hill Chapel Hill USA; ^3^ Department of Chemical and Biomolecular Engineering University of Illinois at Urbana‐Champaign Urbana Illinois USA; ^4^ Department of Chemistry University of Illinois at Urbana‐Champaign Urbana Illinois USA; ^5^ Beckman Institute for Advanced Science and Technology University of Illinois at Urbana‐Champaign Urbana Illinois USA

**Keywords:** chirality, conjugated polymer, liquid crystal, self‐assembly, solvent engineering

## Abstract

Chiral assembly of achiral conjugated polymers has emerged as a new avenue to promote (opto)electronic properties and control the angular momentum of charge carriers and photons. Solvent plays a key role in chiral emergence through the lyotropic liquid crystal (LLC) assembly pathway, yet rules underpinning solvent effect remain elusive. Here, we establish a unifying framework linking solvent quality to aggregate structure and supramolecular chirality. Using redox active conjugated polymers as model systems, we reveal two types of solution aggregation: (1) β1 aggregation, defined by fibrillar structures with weak interchain coupling and torsional backbone, promotes LLC‐mediated chiral assemblies via formation of helical fibers; (2) β2 aggregation, characterized by stacked fibrils with strong interchain coupling and reduced backbone torsion, suppresses chirality. Temperature‐dependent studies show that β2 aggregates revert to β1 upon heating, thus restoring chiral assemblies. Extending to 6 conjugated polymers across 37 polymer–solvent–temperature combinations, we demonstrate the universal link between aggregate type and chiral emergence. Further, chemical doping of chiral films derived from β1 aggregates elevates electrical conductivity by up to 100‐fold across nine polymer–solvent pairs spanning a wide range of processing conditions. These findings reveal general principles for solvent‐guided chiral assembly enabling high‐performance chiral electronics, optoelectronics and spintronics.

## Graphical abstract

Solvent‐dependent aggregation pathways dictate whether conjugated polymers form chiral lyotropic liquid‐crystalline assemblies or achiral packed aggregates. Mapping solvent‐controlled β₁ and β_2_ states reveals that weaker backbone association favors β₁ aggregates and reorganizable twisted mesophases, whereas stronger backbone association favors β_2_ aggregates and tightly packed, non‐chiral assemblies. Chiral β₁ assemblies exhibit higher crystallinity, efficient chemical doping, and enhanced electrical conductivity.

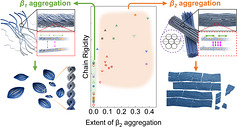

## 1 Introduction

Chirality, a fundamental property describing objects that cannot be superimposed on their mirror images, is observed across all scales of the universe: in subatomic particles such as neutrinos, in biological systems such as human appendages, and in cosmic structures like spiral galaxies. More than a descriptive property, chirality acts as a design principle defined by its inherent selectivity. In biological systems, this handedness underlies the asymmetric biochemical processes that have shaped evolutionary pathways. For example, living organisms primarily use L‐amino acids to build proteins and D‐sugars for energy. This selectivity also governs various in vivo biochemical processes by triggering specific, handedness‐selective immune reactions [1, 2]. Beyond chemistry and biology, chirality has emerged as a critical determinant of function in materials science and device engineering. Its unique selectivity drives advances in several fields, including asymmetric catalysis [3, 4], durable solar cells [5, 6], chemical doping [7], and circularly polarized displays [8]. Furthermore, it is central to the chiral‐induced spin selectivity (CISS) effect, which enables spin‐selective charge transport and is a key area of research for future electronics [9, 10].

Among strategies to introduce chirality in materials, supramolecular chiral assembly of organic systems has received particular attention owing to its tunability and hierarchical character. A particularly promising route is lyotropic liquid crystal (LLC)‐mediated assembly, which produces ordered mesophases with pronounced supramolecular chirality [11, 12]. Unlike approaches that rely on molecular chirality, LLC‐mediated chiral assembly, driven by entropically favorable helix formation [13, 14], allows achiral or weakly chiral building blocks to organize into long‐range coherent chiral structures while retaining solution processability. This combination offers a versatile platform to tailor morphology and function in thin films. The utility of this pathway has been widely demonstrated: harnessing the CISS effect for efficient spin injection and transport [10], extending exciton lifetimes in bulk heterojunctions [12], enhancing efficiency and stability in organic solar cells [15], and boosting doping efficiencies in redox‐active polymers, leading to markedly higher electrical conductivity after chemical doping [7].

Recent work from our group established that LLC‐mediated chiral assembly is a general phenomenon for conjugated polymers, occurring in more than twenty common conjugated polymer systems [13]. This finding significantly broadens the scope of supramolecular chirality in conjugated polymers. Yet, the factors dictating whether a given polymer undergoes LLC‐mediated chiral assembly remain poorly understood. In particular, the role of solvent has not been systematically explored, despite solvent choice being the primary handle for tuning polymer–solvent and polymer–polymer interactions. At present, assembly conditions are identified largely through empirical, trial‐and‐error approaches, underscoring the need for predictive design principles.

Here, we present a systematic study of solvent effects on LLC‐mediated chiral assembly and establish a general framework for determining chiral assembly pathways. We investigate seven oligo(ethylene glycol) (OEG)‐functionalized conjugated polymers across a total of 37 polymer–solvent–temperature combinations. By combining optical techniques such as optical microscopy, UV–vis absorption, and circular dichroism (CD) spectroscopy with structural characterization methods including small‐angle x‐ray scattering (SAXS), atomic force microscopy (AFM), and scanning electron microscopy (SEM), we reveal that the backbone aggregate type of a conjugated polymer is the critical determinant of chiral LLC assembly pathway versus achiral random aggregation. From these results, we derive a matrix linking absorption signatures with assembly outcomes. We further show that chirality in solution transfers directly to the solid state, where films formed through LLC‐mediated assembly exhibit higher crystallinity, as evidenced by grazing‐incidence x‐ray diffraction (GIXD). Finally, we demonstrate that these distinct solid‐state structures strongly influence electronic properties: films prepared via LLC‐mediated chiral assembly achieve substantially enhanced electrical conductivity upon chemical doping, underscoring the central role of solvent‐directed assembly in determining device‐relevant properties.

## 2 Results and Discussion

### 2.1 Solvent Effect on Solution Aggregation and Assembly Pathway

**FIGURE 1:**
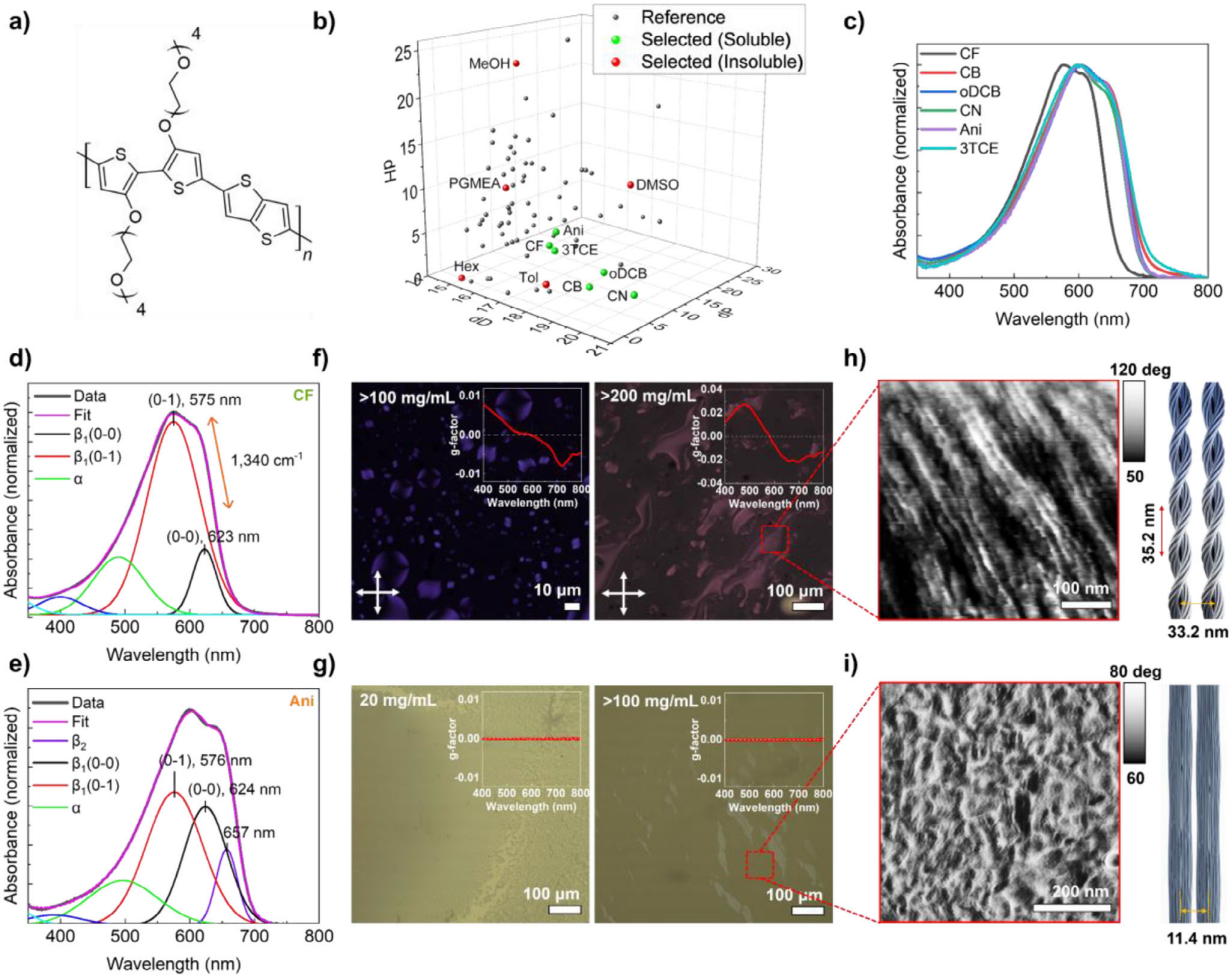
Two distinct solvent‐dependent assembly pathways. (a) Chemical structure of p(g_4_2T‐TT) bearing OEG side chains. (b) Diversity of solvents studied visualized in Hansen Solubility Parameter (HSP) space. Black: reported solvent HSP references in Hansen Solubility Parameters in Practice; green: soluble; red: insoluble. (c) UV–vis absorption spectra of p(g_4_2T‐TT) in selected soluble solvents (5 mg/mL), revealing two aggregation modes. Deconvoluted absorption spectra in (d) CF and (e) Ani, showing α phase (green), corresponding to fully solubilized polymer chains; vibronically correlated β1 aggregation peaks (red and black); and an additional red‐shifted β2 aggregation feature (purple). In all solutions, the α‐phase coexists with aggregated phases. (f) CPOM images of concentrated CF solutions showing birefringent chiral nematic tactoids (left) and coalesced LLC mesophases (right), corresponding to a continuous nematic twist–bend (N_TB_) LC phase. Insets are corresponding CD spectra. (g) Bright field images of concentrated solutions showing aggregates in Ani. AFM images of freeze‐dried concentrated solutions, revealing helical fiber bundles in (h) CF, and orderly stacked fibers in (i) Ani, respectively, with schematic illustration of the aggregates on the right.

We chose to focus our study on bithiophene‐thienothiophene copolymer with tetraethylene glycol side chains, p(g_4_2T‐TT), as the model system to investigate solvent‐dependent chiral LLC emergence (Figure 1a). We will later generalize to other conjugated polymers functionalized with OEG side chains—an emerging class of redox‐active conjugated polymers. These systems exhibit high solubility across a broad range of solvents owing to the high polarity and flexibility of their OEG side chains, which not only enhance polymer–solvent interactions but also suppress strong π–π stacking between polymer backbones [16, 17, 18]. Thus, these systems provide an ideal platform for probing solvent‐dependent assembly behavior. In addition, their unique ion/charge transport characteristics, and biocompatibility positioned them as promising candidates for various applications such as organic electrochemical transistors (OECTs) [16, 17, 18], thermoelectrics [19, 20], electrochromics [21], and so on. Most importantly, we recently found that supramolecular chirality can boost redox reactions [7], and that OEG sidechain is particularly conducive to chiral emergence [13]. Thereby, OEG‐functionalized conjugated polymers represent a particularly compelling class of polymers for uncovering the fundamental principles of solvent‐directed chiral assembly.

We began our investigation by examining the solution‐state aggregation and assembly pathways of p(g_4_2T‐TT) in various solvents. An initial screening of 11 solvents spanning a broad Hansen solubility parameter space was performed to identify suitable candidates (Figure 1b). To evaluate solvent‐dependent solubility, we first determined the critical concentration for phase separation (C_s_) in each solvent, defined as the polymer concentration at which visible aggregation first appeared (Figure S1). Among the eleven solvents screened, five solvents—methanol (MeOH), propylene glycol methyl ether acetate (PGMEA), dimethyl sulfoxide (DMSO), hexane (Hex), and toluene (Tol)—were found to have insufficient solubilities and excluded from further studies. Specifically, these solvents (1) failed to dissolve at polymer concentrations as low as 1 mg/mL and (2) failed to dissolve even at elevated temperatures near the solvent boiling point. In each case, the polymers were either barely dissolved or form large agglomerates (Figure S2). The remaining six solvents—chloroform (CF), chlorobenzene (CB), o‐dichlorobenzene (oDCB), chloronaphthalene (CN), anisole (Ani), and trichloroethylene (3TCE)—could fully solubilize p(g_4_2T‐TT), and were thus selected for further study. The detailed solubility and aggregation analysis are provided in Figures S1 and S2.

Two distinct solvent‐dependent aggregation types were identified using UV–vis spectroscopy (Figure 1c). In CF, the absorption spectra displayed two clear vibronic features at 575 and 623 nm with a 1340 cm^−1^ spacing, characteristic of (0–0) and (0–1) vibronic transitions associated with the π‐π* electronic transition [22, 23]. Peak deconvolution unveils a hidden featureless band at 493 nm which we assign to dispersed polymer chains with minimal intra‐ nor inter‐chain electronic coupling (Figure 1d). The presence of a vibronically structured low‐energy band suggests the formation of J‐aggregates or intra‐chain electronic coupling likely induced by inter‐chain aggregation (later confirmed by SAXS). A relatively low (0–0)/(0–1) peak ratio indicates that this aggregated state possesses significant torsional disorder in the conjugated backbone limiting the extent of J‐aggregation [23, 24]. We designate this aggregation type as the β1 phase [25]. By contrast, spectra in CB, oDCB, CN, Ani, and 3TCE were broadened toward the low‐energy region corresponding to a red‐shifted onset of absorption. From peak deconvolution (Figure 1e; Figures S3 and S4), we determined that this spectra shift is due to the emergence of an additional peak at 657 nm that was not vibronically related to the (0–0) nor (0–1) peak. Comparison between the α + β1 and α + β1 + β2 fitting models showed that the β1‐only model cannot reproduce the 650–660 nm absorption feature, supporting the assignment of this additional low‐energy component (Figure S5). A detailed peak‐fitting protocol and mathematical justification are provided in the Methods section. The emergence of this low‐energy band reflects enhanced exciton delocalization due to stronger interchain interactions or H‐aggregation [25]. Furthermore, these spectra exhibited a higher (0–0)/(0–1) ratio, consistent with a less torsional backbone conformation likely induced by H‐aggregation distinct from the β1 phase [25]. We designate this new H‐aggregate as the β2 phase. Structural evidence supporting these assignments will be provided in later sections.

Using our standardized drop‐and‐dry procedure [12] (see Methods section) combined with optical microscopy and CD spectroscopy, we found that the two distinct solution aggregation modes led to markedly different assembly pathways driven by concentration increase relevant to the evaporative assembly process during solution processing. To ensure the reliability of our observations, two precautions were taken: (1) equilibrium was achieved through repeated slow thermal annealing followed by 30 min of aging at the target temperature (Figure S6), and (2) contributions from linear birefringence and linear dichroism were carefully minimized by the 4‐scan method [26]. In CF solutions with β1 aggregation, increasing concentrations exceeding 100 mg/mL produced microscale chiral tactoids with strong birefringence under cross‐polarized optical microscope (CPOM) (Figure 1f, left). The corresponding CD spectra showed clear bisignate feature from the Cotton effect [27] indicating excitonic coupling likely associated with β1 aggregation. The dissymmetric factor for absorption (g‐factor) reached ∼0.01 at ∼100 mg/mL, further increasing concentrations to >200 mg/mL increased the g‐factor to almost 0.03 (Figure 1f, right). In contrast, for solutions containing β2 aggregates, only large agglomerates were observed upon increasing concentration above 20 mg/mL via transmission bright‐field optical microscopy, with no birefringence nor CD response (Figure 1g).

Next, AFM imaging on freeze‐dried solution samples resolved nanoscale features, providing insight into the structural basis to two distinct types of solution aggregation. The solution with β1 aggregation contains helical fiber bundles with a half‐pitch (*p*_1/2_) length of 35.2 ± 5.4 nm and a lateral stacking distance of 33.2 ± 5.5 nm (Figures 1h and S7). These fibers exhibit high orientational order locally, consistent with the strong birefringence observed under CPOM. By contrast, β2 solution displayed orderly stacked fibers with stacking distance of 11.4 ± 1.9 nm absent from signatures of helicity explaining zero CD signal (Figures 1i and S8). Although short range order is observed in fiber stacking at the nanoscale, there is lack of long‐range order, consistent with the absence of birefringence.

To further examine whether the relative β1/β2 aggregate population changes upon increasing concentration, we performed concentration‐dependent UV–vis measurements for CF and Ani solutions from 10 to 200 mg mL^−^^1^ (Figure S9). The characteristic spectral features associated with β1 and β2 aggregation were largely preserved across the entire concentration range. In particular, no new β2‐specific, red‐shifted component or systematic increase in the β2 population was observed with increasing concentration. Instead, the spectra became progressively broadened with only minor peak shifts. These results indicate that increasing concentration does not significantly alter the primary aggregate species or their relative population. Rather, concentration increase promotes higher‐order mesophase development from pre‐existing primary aggregates, leading to broader distributions of local packing environments and enhanced intermolecular electronic coupling.

**FIGURE 2:**
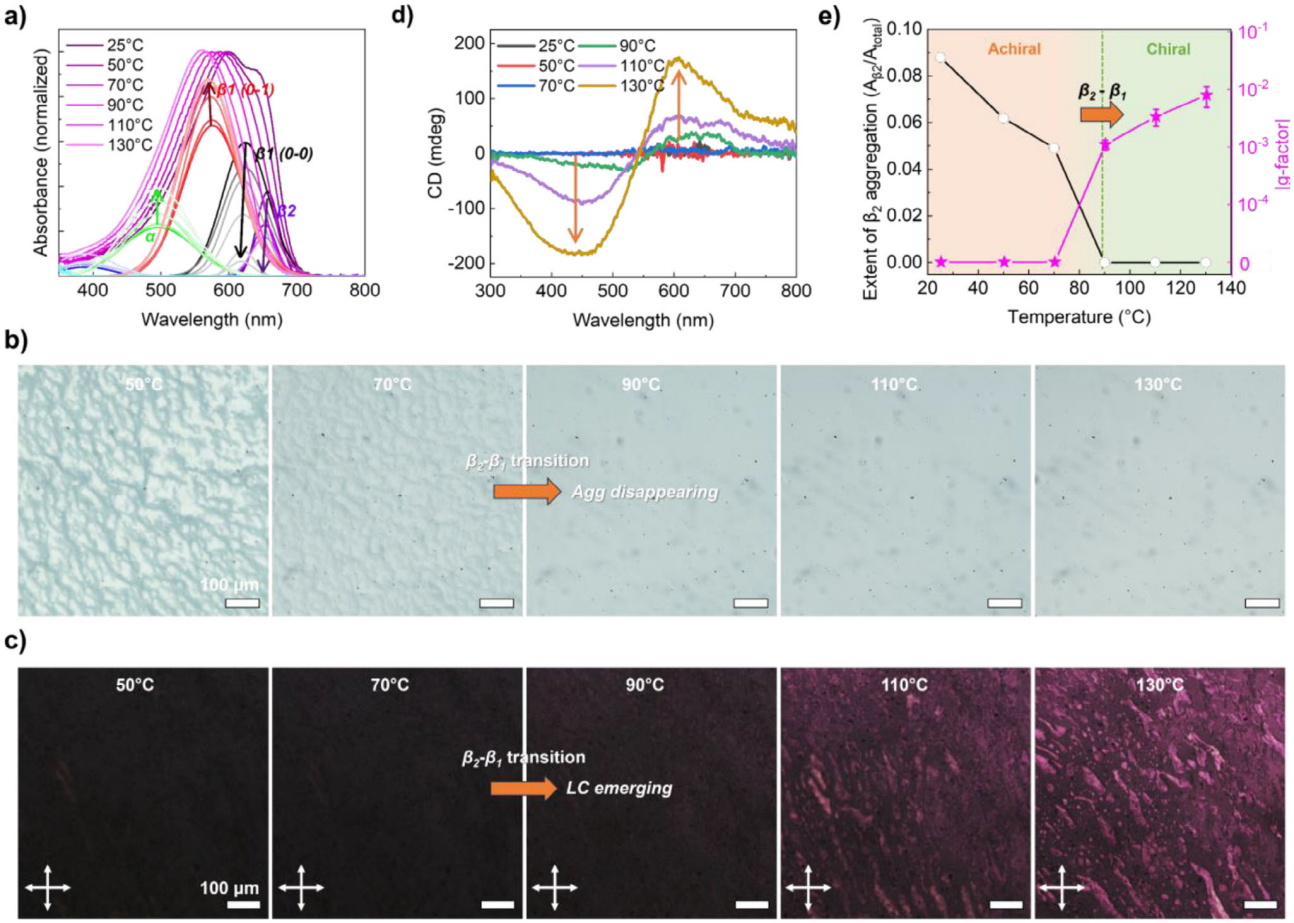
Thermally activated transition between β1 and β2 aggregates. (a) Deconvoluted UV–vis absorption spectra of p(g_4_2T‐TT) in Ani (5 mg/mL) at different temperatures, showing the progressive disappearance of the β2 peak (purple) and decreasing (0–0)/(0–1) ratio associated with β1 aggregation. (b) Transmission bright‐field optical images of 20 mg/mL Ani solutions, showing β2 aggregates at 50°C–70°C and their disappearance above 90°C. (c) CPOM images of concentrated Ani solutions (>200 mg/mL) over the same temperature range, showing the emergence of LLC textures and birefringence above 90°C, which became more prominent with increasing temperature. (d) Temperature‐dependent CD spectra of concentrated Ani solutions (>200 mg/mL), with the onset of bisignate CD signals at 90°C and steady growth up to 130°C. (e) Quantitative analysis of β2 aggregate content (black, left axis) and g‐factor (magenta, right axis) as a function of temperature, showing that suppression of β2 aggregation coincides with the emergence of chirality. All temperature‐dependent measurements were performed after heating–cooling cycles, and this thermally activated transition between two aggregates is reversible.

We hypothesize that the distinct assembly states originate mainly from differences in backbone aggregation defined by solvent quality. In solvents with β2 aggregation, polymer backbones have lower solubility and thus exhibit more planar conformation and stronger interchain aggregation, promoting crystallization‐driven assembly pathway upon concentration increase/solvent evaporation. In solvents with β1 aggregation, higher backbone solubility leads to weaker interchain aggregation while maintaining intrachain aggregation; weakly associated polymer fibers are conducive to forming LLC rather than directly crystallize. We previously showed that such LLC pathway is conducive to chiral emergence from achiral conjugated polymers [13, 14]. To validate this hypothesis, we varied the solution temperature to modulate the solvent quality and polymer solubility. UV–vis measurements in a β2‐type solvent Ani showed that at elevated temperatures (increased solubility), the β2 aggregate progressively diminished and dissolved entirely ∼90°C, promoting a transition from β2 to β1 aggregation (Figures 2a and S3). Also, upon heating, the intensity of the α peak increased while the (0–0)/(0–1) vibronic ratio decreased, consistent with enhanced solubility and increased backbone torsion. Temperature dependent bright‐field images of 20 mg/mL Ani solutions confirmed this transition: β2 aggregation features vanished near 90°C, in agreement with the UV–vis results (Figure 2b). Temperature‐dependent CPOM and CD measurements of concentrated Ani solutions (>200 mg/mL) further provided direct evidence of this transformation (Figure 2c, and 2d). At 90°C, when β2 aggregates fully dissolved, LLC phases emerged giving rise to CD signals. With further heating upto 130°C, both the LLC features and the g‐factor increased progressively. Quantitative analysis of β2 aggregate content (defined as the ratio of β2 peak area to total area under the UV–vis absorption curve) and the corresponding g‐factors is summarized in Figure 2e. We note that g‐factor analysis in thin samples can be affected by artifacts arising from anisotropy and scattering, particularly in spectral regions with negligible absorption [28, 29]. To minimize these effects, all reported g‐factor values were extracted only from the absorption‐active spectral region using the protocol summarized in Figure S10.

Importantly, the temperature‐mediated β2 ↔ β1 transition is fully reversible upon cooling, as demonstrated by the reversibility tests summarized in Figure S11. Furthermore, CF, which forms β1 aggregates at room temperature, also undergoes a transition toward the β2 state upon cooling to −20°C, further supporting that the aggregation state is governed by temperature‐dependent solvent quality (Figure S12).

We further verified that aggregation is primarily governed by backbone interactions rather than side‐chain packing through solution wide‐angle x‐ray scattering (WAXS) experiments (Figure S13a). In CF, where the polymer forms the β1 state and subsequently chiral LLC assembly, the solution WAXS pattern shows no clear evidence of tightly ordered packing of sidechain and backbone. In contrast, in Ani, where the polymer forms the β2 state and does not lead to chiral LLC assembly, a distinct π–π packing feature is observed. This result supports our assignment that β2 corresponds to a more tightly backbone‐packed aggregate state, whereas β1 represents a “loosely” associated aggregate conducive to LLC formation. Importantly, characteristic OEG side‐chain packing features, which have been reported near q ≈ 1.4 Å^−^^1^, are not clearly observed under either condition [30]. We attribute this absence to the high solubility and miscibility of OEG side chains in the solvents examined. To further assess this point, we used poly(ethylene glycol) dimethyl ether as a molecular proxy that shares structural and chemical similarity with the OEG side chains (Figure S13b). All solvents tested exhibited high miscibility with this model compound up to 50 vol%, supporting the conclusion that, for the OEG‐functionalized conjugated polymers investigated in this work, solvent‐dependent aggregation primarily arises from backbone interactions rather than OEG side‐chain aggregation.

We note that this inference does not invalidate the importance of OEG side chains in the LLC assembly process. Previous studies on lyotropic liquid‐crystalline conjugated polymers suggest that LLC formation requires an intermediate state of aggregation, where polymer chains are sufficiently associated to form anisotropic ordered aggregates but still retain sufficient mobility to reorganize into a liquid‐crystalline mesophase [31, 32, 33]. When aggregation becomes too strong, mesophase can be lost: tight backbone packing can promote a crystallization‐mediated pathway, while strong side‐chain stacking can restrict chain mobility and suppress mesophase development, leading to disordered aggregates [31]. The highly solvated and flexible nature of OEG side chains can help mitigate such unfavorable side‐chain stacking [30], thereby enabling LLC‐mediated assembly providing adequate backbone solubility.

This interpretation is supported by control experiments using non‐OEG‐substituted conjugated polymers. PBTTT‐C14 and P3HT did not form chiral LLC phases across a broad range of solvent conditions, but instead produced featureless sheet‐like morphologies at high concentrations (Figure S14). These results highlight the critical role of OEG side chains in enabling the chiral LLC assembly pathway. However, within the OEG‐functionalized polymer systems studied here, the OEG side chains appear sufficiently solvated and do not exhibit distinct ordered side‐chain packing. Thus, we interpret that the β1 versus β2 distinction is primarily dictated by effective backbone solubility and backbone–backbone interactions.

### 2.2 Characterization of Solution Aggregate Structures

**FIGURE 3:**
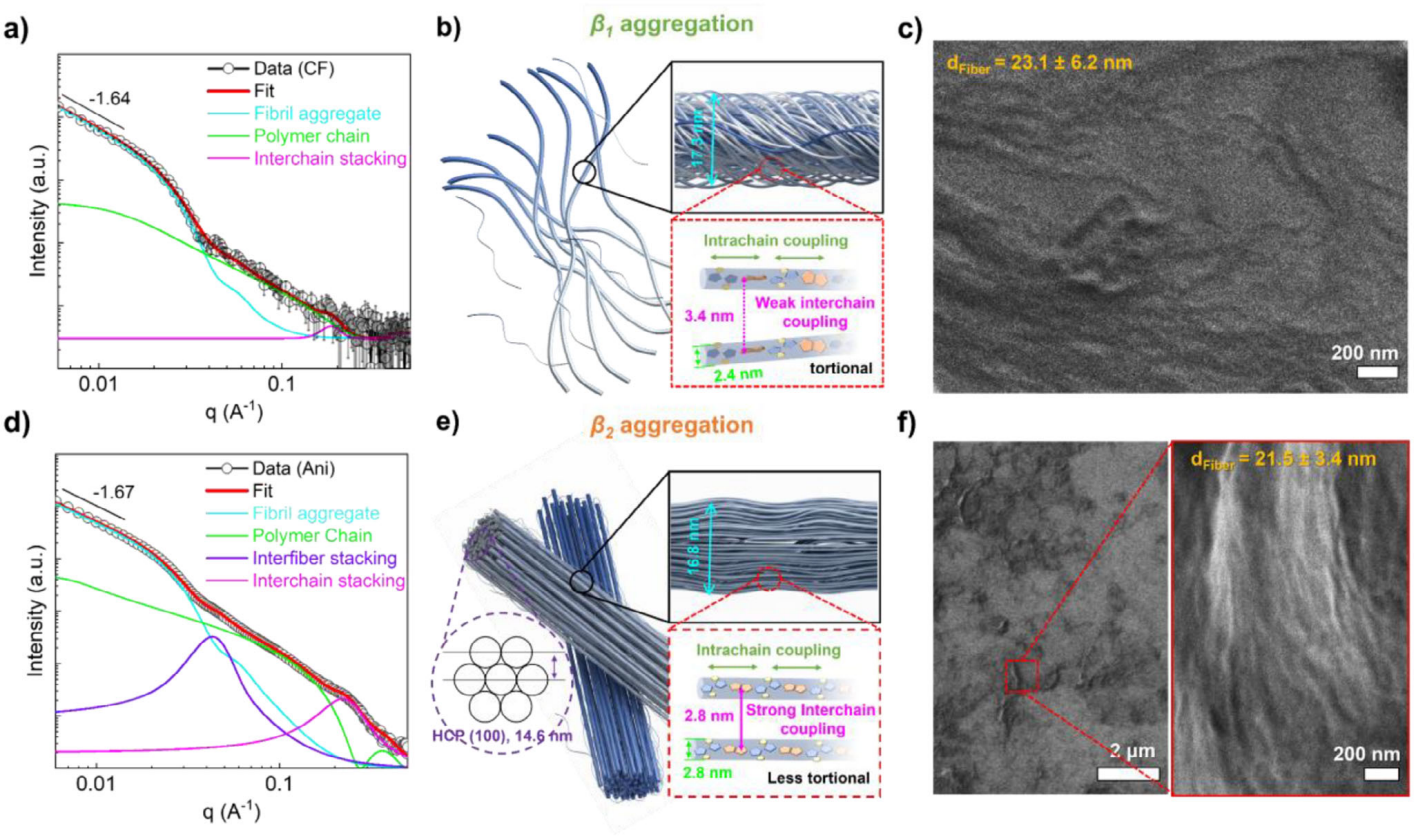
Analysis of solvent‐dependent solution aggregate structures. (a) SAXS profile of β1‐type solutions (5 mg/mL, CF) fitted with a two‐flexible‐cylinder model plus a weak pseudo‐Voigt peak, consistent with loosely coupled fibrillar aggregates. (b) Schematic illustration of β1 aggregate structure. (c) SEM image of freeze‐dried β1 solutions, revealing small loosely associated fibrillar aggregates. (d) SAXS profile of β2‐type solutions (5 mg/mL, Ani) fitted with a two‐flexible‐cylinder model and two pronounced pseudo‐Voigt features, indicating strong interchain coupling and highly ordered interfiber packing. (e) Schematic illustration of β2 aggregate structure. (f) SEM images of freeze‐dried β2 solutions, showing sub‐micron aggregates composed of tightly packed fibrils.

We next characterized the structures of the β1 and β2 solution aggregates to validate the assembly mechanism proposed above, by performing SAXS, SEM and AFM analyses on 5 mg/mL solution samples. For the β1‐type solution, SAXS revealed the presence of fibrillar aggregates [34] as evidenced by a Guinier knee in the low‐q region (q < 0.05 Å^−^^1^), corresponding to the cross section of the fibrils (Figure 3a). The scattering profile was well described by a two‐flexible‐cylinder model along with an additional pseudo‐Voigt peak [34]. In this case, individual polymer chains (diameter = 2.4 ± 0.2 nm, green) assembled into larger fibrils (diameter = 17.3 ± 0.4 nm, cyan) (Figure 3b). The slope in the low‐q region (<0.05 Å^−^^1^) is in the range of −1.6 to −1.7, indicating the dimensionality of the fiber network is between 1 (rod‐like) and 2 (sheet‐like). The presence of a possible pseudo‐Voigt peak (magenta) was assigned to interchain stacking within the aggregated fibrils, with a packing distance of 3.4 ± 0.3 nm. However, the intensity of the pseudo‐Voigt peak is barely above the noise level suggesting weak interchain stacking if at all. This is consistent with absence of the H‐aggregate peak at 657 nm from UV–vis spectroscopy. SEM imaging of freeze‐dried solution samples provided structural evidence supporting the model, revealing fibrillar aggregates with average sizes of 23.1 ± 6.2 nm, in reasonable agreement with the SAXS analysis (Figure 3c).

In contrast, β2 aggregates displayed distinct new structure factor peaks, indicating additional interfibril stacking not present in β1 aggregates (Figure 3d). The scattering profile could also be fitted with the two‐flexible‐cylinder model for the aggregate and solubilized polymer chains, yielding fibril aggregate diameters of 16.8 ± 1.3 nm and polymer diameters of 2.8 ± 0.1 nm. The pseudo‐Voigt peak for lamella stacking internal to the fibril aggregate was markedly stronger than in the β1 aggregate returning a 2.8 ± 0.1 nm stacking distance. More importantly, a new structure factor peak appeared at 0.43 Å^−^^1^ and was fitted to a pseudo‐Voigt peak (purple) giving a stacking distance of 14.6 ± 0.1 nm. We ascribe this feature to spacing between (100) planes of a hexagonal close‐packed (HCP) lattice of fibrillar aggregates with individual fiber diameter of ∼16.7 nm. This result suggests that fibrils of polymer aggregates further assemble into higher‐order superstructures (Figure 3e). Ordered interchain and interfiber stacking in β2 aggregates is reflected at the molecular scale, where the persistence length of individual polymers was 41.6 ± 2.2 nm in β2 aggregates, substantially larger than that (7.0 ± 2.5 nm) in β1 aggregates, indicating far greater backbone planarity possibly arising from strong interchain interactions. All detailed fitting parameters of SAXS are summarized in Tables S1 and S2. These conclusions are strongly supported by SEM imaging of freeze‐dried β2 samples, which revealed submicron‐scale aggregates (Figure 3f, left) composed of tightly packed fibrils with characteristic sizes of 21.5 ± 3.4 nm (Figure 3f, right), in good agreement with SAXS analysis.

All the above characterizations culminate in a unified mechanism for solvent‐dependent assembly pathways. In solvents with strong polymer–solvent interactions or high solubility, β1 aggregation is favored, characterized by weak interchain backbone aggregation and high flexibility and torsional freedom. Weakly associated polymer fiber aggregates have sufficient degrees of freedom to assemble into LLC upon increasing concentration. Chiral helical structure emerges in the LLC phase to minimize excluded volume and to reach the densest packing [13, 14]. By contrast, in solvents conducive to strong polymer–polymer interactions or low solubility, β2 aggregation emerges. Here, reduced solubility drives backbone aggregation and restricts torsional freedom, resulting in direct crystallization into achiral assemblies characterized by highly ordered interchain and interfiber packing.

More importantly, the β1 and β2 aggregation modes can be reversibly interconverted by modulating polymer solubility through temperature control, as we observed in Figure 2. Upon heating, β2 aggregates progressively convert to β1 aggregates, restoring torsional freedom and enabling the emergence of LLC chiral phases. High‐temperature SAXS measurements of Ani solutions further confirmed this transformation, revealing scattering profiles that closely resembled those of β1‐type CF solutions upon heating to 130°C. Specifically, the pseudo‐Voigt peak in the mid‐q range (0.04–0.05 Å^−^^1^) disappears with only a very weak pseudo‐Voigt peak at the high‐q range (∼0.2 Å^−^^1^) that remains (Figure S15 and Table S3). This reversible transition underscores that solubility of the backbone is the determining factor dictating whether conjugated polymers assemble into chiral LLC structures from semiflexible fiber aggregates or crystallize into superlattices comprised of achiral fibers.

### 2.3 Impact of Aggregate Type on Film Morphology

**FIGURE 4:**
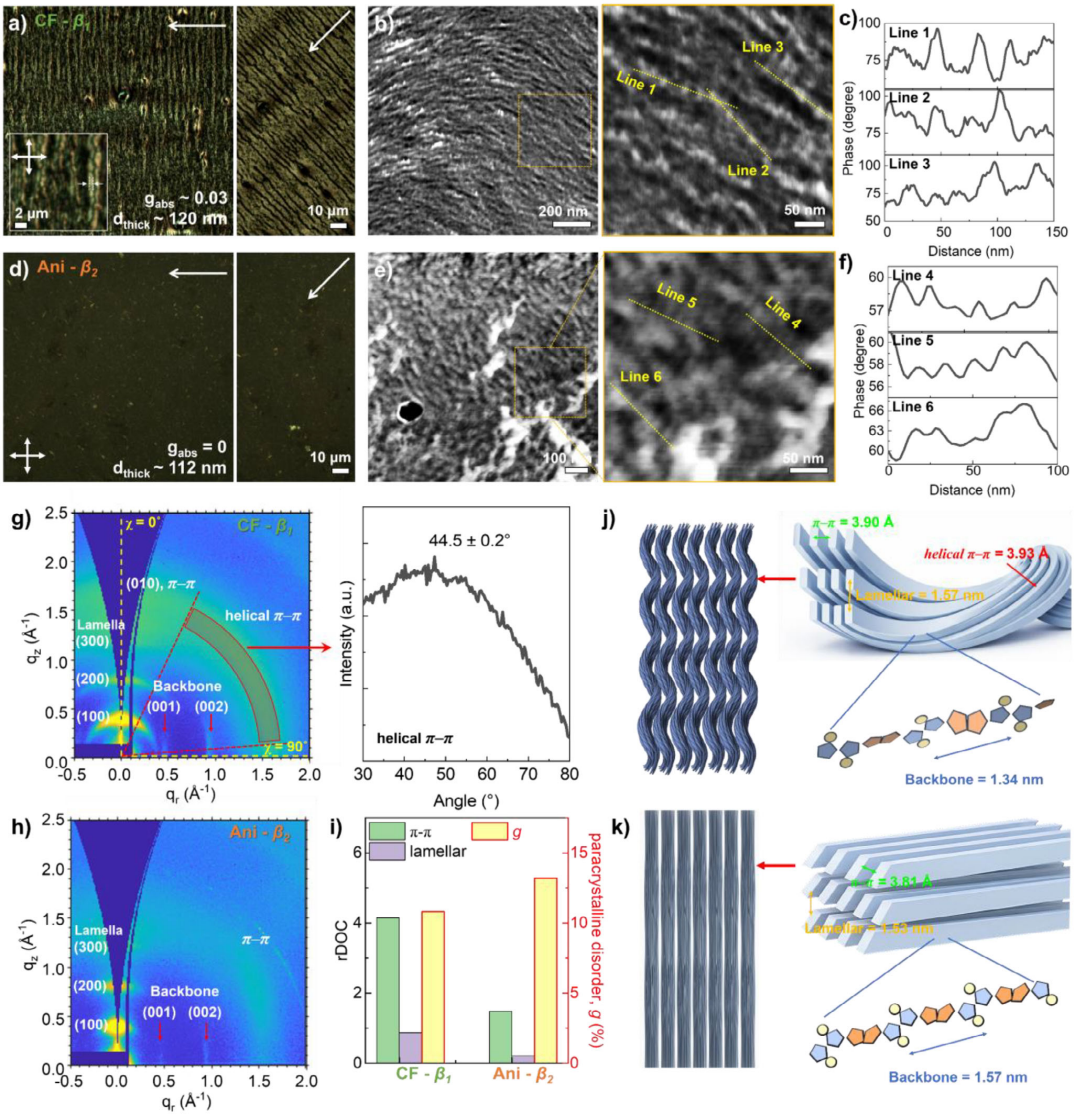
Impact of aggregate type on film morphology. (a) CPOM and (b) AFM phase images of p(g_4_2T‐TT) films blade‐coated from CF (β1 aggregation), showing twin domains with helical fibers. (c) Height profile obtained from line‐cuts indicate a half‐pitch (*p*_1/2_) length of 24.3 ± 6.5 nm. (d) CPOM and (e) AFM phase images of films bladed coated from Ani (β2 aggregation), showing weak birefringence and orderly stacked fibers. (f) Height profiles obtained from line cuts reveal a fiber stacking distance of approximately 16.4 ± 2.5 nm, with no detectable chirality. The thickness of each film is 120 nm and 110 nm for the β1 and β2 films, respectively. 2D GIXD (parallel) patterns of (g) β1 and (h) β2 films, respectively. In β1 films, q‐χ plot in the χ‐range of 25°–80° and *q*‐range of 1.5–1.8 Å^−^^1^ (highlighted in red in the GIXD image) shows a helical π–π stacking peak at χ=44.5° and *q* = 1.60 Å^−^^1^. (i) Quantitative comparison of rDoC and paracrystalline disorder (*g*) of (100) peak, highlighting the superior crystallinity of films with β1 aggregation (CF). Schematics depicting molecular packing in (j) β1 and (k) β2 films.

We next investigate how aggregate structure in the solution defines the morphology of bladed‐coated films. We chose to perform blade coating in the evaporation regime at low coating speeds (see Methods section) to approximate the near‐equilibrium, concentration‐driven assembly pathway evaluated above, while ensuring reproducible film formation (Figure S16). This coating regime successfully transferred the supramolecular chirality observed in solution to the solid state, as confirmed by CD measurements (Figure S17). Image analysis revealed markedly different film morphology depending on the aggregate type. In films blade‐coated from solutions with β1 aggregates, clear twin domains with average domain width of 750 ± 187 nm were observed under CPOM (Figure 4a), a hallmark of supramolecular chirality arisen from twist‐bent nematic mesophases [12]. AFM analysis of these domains revealed helical fibers 27.9 ± 10.4 nm in interfiber distance and 24.3 ± 6.5 nm in half‐pitch (*p*_1/2_) length (Figure 4b,c, and S18), consistent with the LLC structures identified in concentrated solutions (Figure 1). By contrast, films blade‐coated from solutions with β2 aggregates exhibited weaker birefringence under CPOM, without clear domain features (Figure 4d). AFM imaging of these regions (Figure 4e,f) revealed densely and orderly stacked fibers at 16.4 ± 2.5 nm spacing, comparable with the fiber spacing of 11.4 ± 1.9 nm in concentrated solution (Figure 1). The strong birefringence observed in β1‐derived films, compared with the weak optical response of β2‐derived films, indicates a higher degree of crystallinity and/or molecular alignment. We further analyzed polymer packing at the molecular scale using GIXD. The highly crystalline conjugated polymer films exhibit sharp out‐of‐plane lamellar diffraction peaks at approximately 0.4, 0.8, and 1.2 Å^−1^, along with well‐defined in‐plane backbone and π–π stacking peaks at ∼0.47, ∼0.95, and ∼1.61 Å^−1^, respectively (Figures 4g,h and S19). In films coated from CF (β1 aggregates), we observed a diffraction ring at q_r_ = 1.60 Å^−^^1^ with maximum intensity centered around 45°. We attribute this feature to helical π–π stacking, a motif that we reported previously in a variety of structurally chiral polymer systems [7, 35]. In contrast, films coated from Ani (β2 aggregates) has a more focused π–π stacking peak at *q*_r_ = 1.65 Å^−^^1^ with intensity centered at *χ*∼ 60°. We attribute this diffraction signature to “edge‐on”, brick‐work like (rather than helical) π–π stacking but with the polymer backbone tilted with respect to the substrate rather than perfectly edge‐on. Such backbone tilt is not uncommon in highly crystalline polymer systems [36]. Quantitative analysis confirmed that β1 chiral films exhibited a slightly expanded π–π distance of 3.90 Å, compared to 3.81 Å for β2 achiral films. Similarly, the out‐of‐plane lamellar stacking distance decreased from 1.57 nm in β1 films to 1.53 nm in β2 films, reflecting tighter packing in β2 aggregates. Further, the lamellar peaks in β1 films exhibit broader orientation distribution, compared to more focused lamellar peaks from β2 films. This highlights two distinct packing motifs: β1 films feature chiral helical assemblies naturally leading to broad orientation distribution of both lamellar and π–π stacking peaks due to “spiraling” of stacked polymers around the chiral axis; β2 films instead has tightly packed, highly oriented crystallites with conventional brickwork packing.

Despite larger stacking distances, β1 films demonstrated markedly higher relative degrees of crystallinity (rDoC) measured via both lamellar and π–π stacking peaks. Consistently, the paracrystalline disorder of the (100) lamellar peak was also smaller in β1 films (Figure 4i). Specifically, the π–π stacking rDoC was 4.15 for β1‐derived films and 1.48 for β2‐derived films, while the lamellar stacking rDoC was 0.87 and 0.21, respectively. In addition, β1‐derived films exhibited a lower paracrystalline disorder of 10.8% for the (100) lamellar peak, whereas β2‐derived films showed a higher disorder of 13.2%. These trends are consistent with the stronger birefringence and macroscopic optical anisotropy observed in β1‐derived films under CPOM which are highly reproducible (Figure S16). All quantitative GIXD peak‐fitting results for both films are summarized in Table S4. A proposed molecular stacking model in the fibril aggregate is shown in Figure 4j,k.

What led to higher crystallinity in β1 films when the molecular packing is “looser” than the β2 aggregate? We propose that it is LLC assembly pathway that ultimately led to higher crystallinity in blade‐coated films, not the internal structure of the fibers. Solvents that promote β1 aggregation provide sufficient backbone flexibility and torsional freedom, allowing chains to aggregate into helical fibers that give rise to chiral LLC assembly pathway conducive to long range order and high degree of crystallinity upon solidification. In contrast, solvents that drive β2 aggregation enforce strong backbone aggregation and tightly packed fibrillar structures with restricted torsional freedom. Although tightly packed, strong inter‐fiber stacking prevents formation of LLC and chiral emergence during blade coating as to favor direct crystallization into kinetically trapped achiral assemblies compromising the overall degree of crystallinity. Indeed, we have shown recently that chiral helical assemblies are more thermodynamically stable than achiral crystalline structures leading to higher degree of crystallinity and overall denser films [7].

### 2.4 Generality of Solvent Effect on Assembly Pathway

**FIGURE 5:**
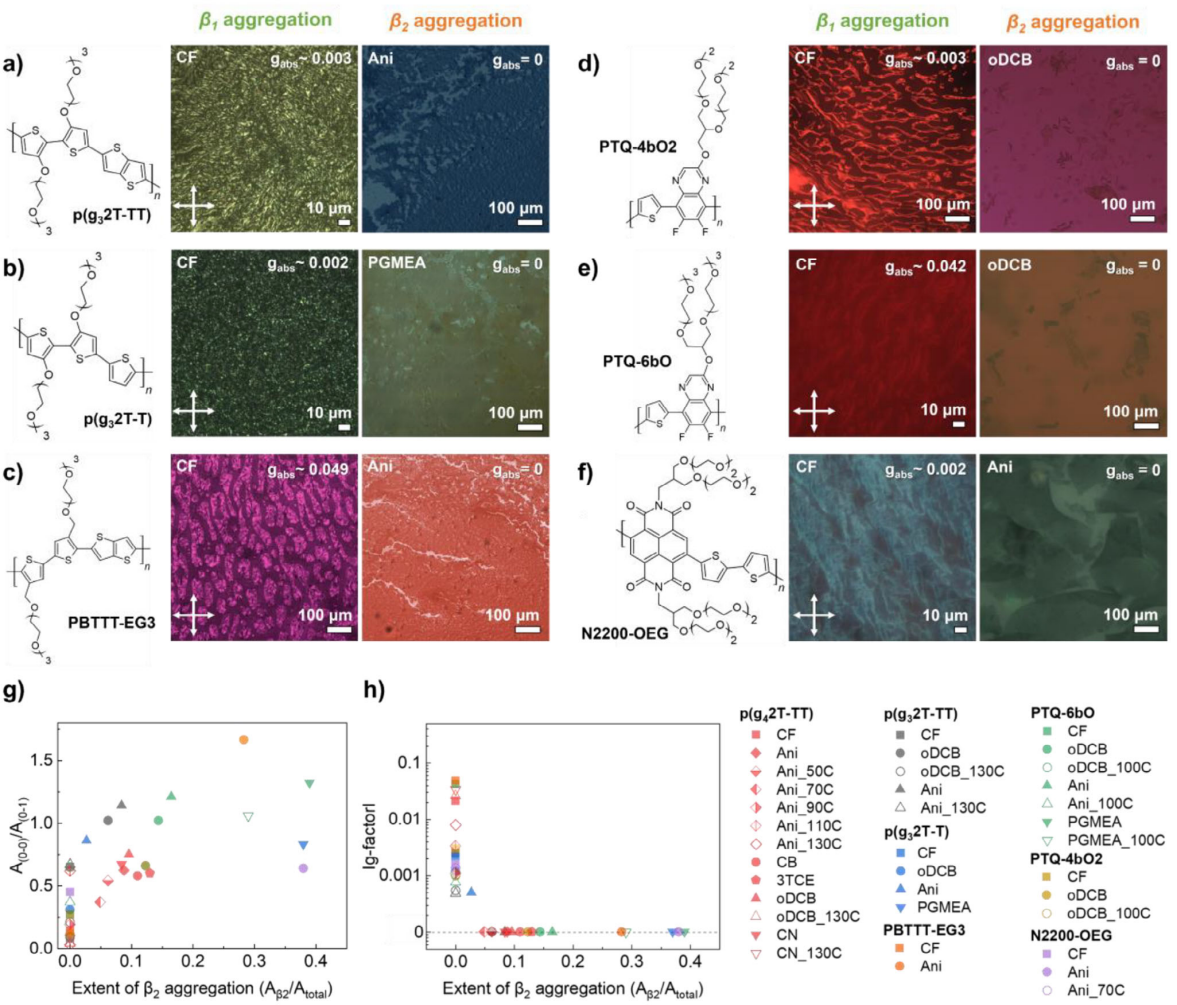
Generality of solvent‐dependent solution aggregation and chiral emergence across multiple conjugated polymer systems. (a–f) Chemical structures of six additional OEG‐functionalized conjugated polymers and CPOM images of concentrated solutions in β1‐ (∼200 mg/mL) and β2‐type (∼100 mg/mL) solvents. Each Inset shows corresponding g‐factor. In all cases, β1‐type aggregation produced chiral LLC phases, whereas β2‐type aggregation led to achiral assemblies. (g) Quantitative analysis of β2 aggregate content and (0–0)/(0–1) ratio across the polymer library, showing that higher extent of β2 aggregation is correlated with higher (0–0)/(0–1) ratio, suggesting more planar backbone. (h) Dependence of g‐factor on extent of β2 aggregation, demonstrating that suppression of β2 aggregation coincides with chiral emergence.

We next investigated whether the solvent‐dependent aggregation and assembly behavior is transferable to other polymer systems. Toward this aim, we synthesized six additional conjugated polymers bearing OEG side chains with backbone structures spanning thiophene (Figure 5a–c), quinoxaline (Figure 5d,e), and naphthalene diimide (Figure 5f) units. In all cases, OEG side chains were selected to afford testing of a broad range of solvents. Remarkably, all six polymers exhibited consistent solvent‐dependent assembly pathways, with solvents containing β1 aggregates giving rise to chiral liquid crystals, and solvents with β2 aggregates yielding achiral aggregation only upon increasing solution concentration (Figure 5a–f).

To substantiate this observation, we used UV–vis and CD spectroscopy with temperature variation to quantify three key parameters: (1) the extent of β2 aggregation, (2) the (0–0)/(0–1) ratio as an indicator of backbone planarity and solubility, and (3) the g‐factor as a measure of chirality. Across the polymer systems, we observed a correlation between (0–0)/(0–1) ratio and the extent of β2 aggregation in solution, with higher (0–0)/(0–1) ratios corresponding to higher extent of β2 aggregation. This suggests that solvents with lower solubility and higher backbone planarity are conducive to β2 aggregation which features highly ordered intra‐ and inter‐chain stacking (Figure 5g). More importantly, CD analysis confirmed the clear link between aggregate type and chirality (Figure 5h). When strong β2 aggregation was present in dilute solutions, no chiral liquid crystals were observed upon concentration increase. Conversely, selecting solvents or increasing temperature to suppress or dissolve β2 aggregate led to emergence of chiral liquid crystal phases with measurable g‐factors reaching values exceeding 0.1. Together, these results demonstrate that the observed solvent‐dependent assembly pathways can be generalized to a range of conjugated polymers, solvent systems and temperature conditions, all governed by type of backbone aggregation. Full supporting data and analyses are provided in Supporting Information, including UV–vis analysis (Figures S3 and S4), CD measurements (Figures S20–S23), CPOM images demonstrating LC phases in β1 solvents (Figure S24).

### 2.5 Impact of Aggregate Type on Chemical Doping

Finally, we investigate how aggregate type impacts conductivity in chemically doped devices. In our previous work, we found that supramolecular chirality facilitates chemical doping, owing to the combined high crystallinity and the CISS effect, which together lead to increased doping efficiency in chiral structures [7]. However, such phenomenon has not been linked to aggregate type in our previous work. Since our model polymers contain OEG side chains, which are well known for enabling efficient ion transport [18, 19, 20] and chemical doping [37, 38], we believe understanding the role of aggregate structure on doping is of practical relevance to potential device applications.

**FIGURE 6:**
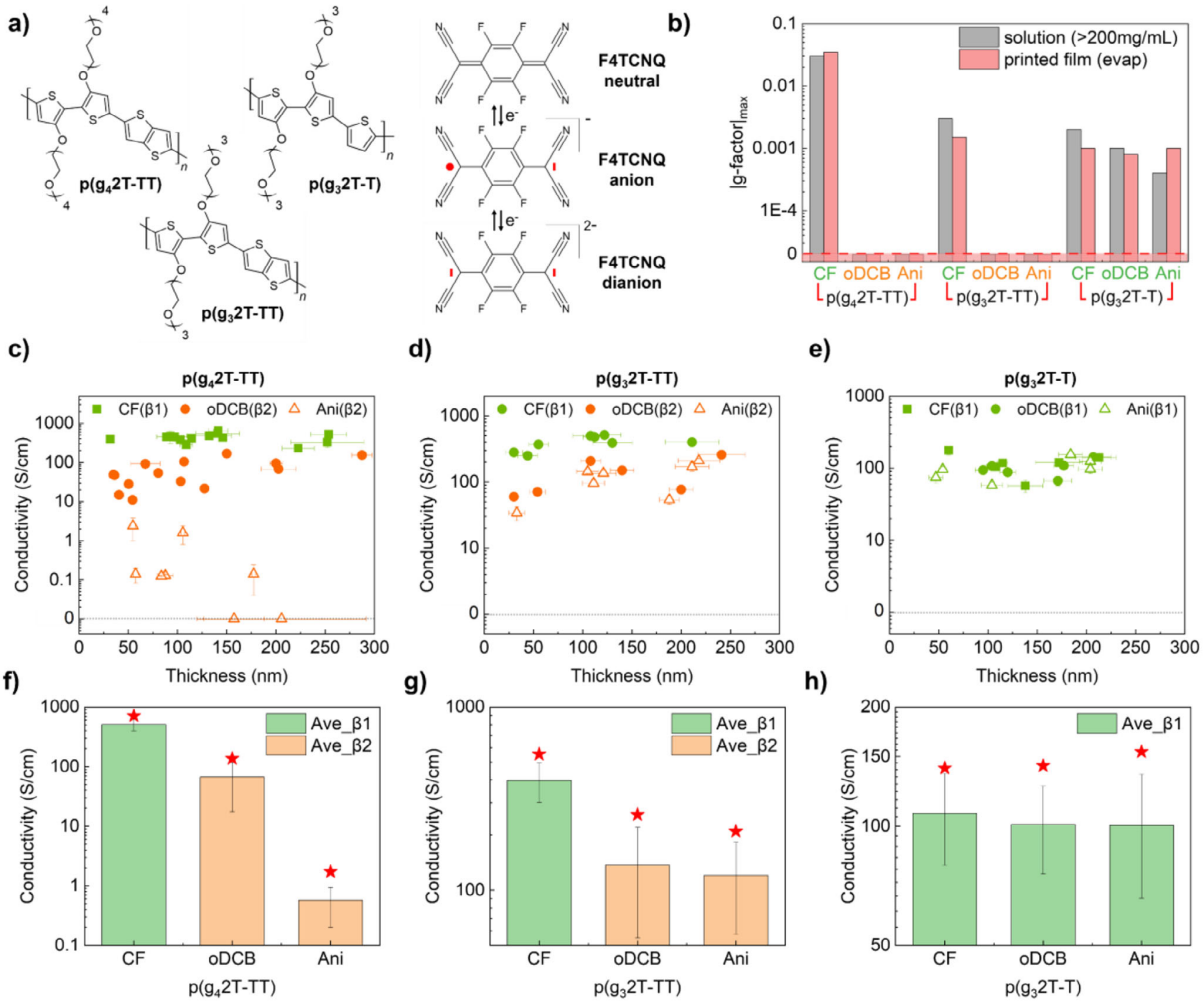
β1 aggregates promote conductivity upon chemical doping. (a) Chemical structures of three representative OEG‐functionalized conjugated polymers (p(g_4_2T‐TT), p(g_3_2T‐TT), and p(g_3_2T‐T)) and F4TCNQ dopant, highlighting its electron‐withdrawing mechanism via anion and dianion formation. (b) g‐factors of films fabricated from different solvents compared to those from concentrated solutions, measured by CD. Films prepared from β1‐type solvents (green) exhibit supramolecular chirality, whereas those from β2‐type solvents (orange) do not. Average conductivity (bars) and maximum conductivity (red stars) of doped films for (c) p(g_4_2T‐TT), (d) p(g_3_2T‐TT), and (e) p(g_3_2T‐T), showing that β1‐type solvents (green) consistently yield superior conductivity compared to β2‐type solvents (orange). Error bars indicate the standard deviation calculated from measurements of more than six individual films, with 50 measurements performed on each film. Conductivity of chemically doped β1‐ and β2‐type films with varying thicknesses for three polymers: (f) p(g_4_2T‐TT), (g) p(g_3_2T‐TT), and (h) p(g_3_2T‐T). Green‐labeled solvents correspond to β1 conditions, producing chiral films, while orange‐labeled solvents correspond to β2 conditions, producing achiral films. The red star indicates the maximum conductivity.

Among the polymer library we investigated, only thiophene‐based polymers exhibited measurable doped conductivity, whereas quinoxaline‐ or naphthalene diimide‐based polymers showed no detectable doped conductivity (see details in Method section). Specifically, we selected F4TCNQ, a p‐type dopant that withdraws electrons via anion and dianion formation (double‐doping), leading to efficient chemical doping (Figure 6a) [39, 40]. Doping was performed using an incremental concentration method, which has been reported to achieve high conductivity by minimizing structural disorder during the doping process [41]; additional experimental details are provided in the Methods section. All films were fabricated by blade coating under the evaporation regime using three different solvents, CF, oDCB, and Ani, for each polymer. We confirmed that supramolecular chirality from β1‐type solvents was successfully transferred into the thin films, while films coated from β2‐type solvents preserved non‐chiral assemblies from the solution (Figure 6b).

We found that the conductivity of doped films strongly depended on the aggregate structure. For p(g_4_2T‐TT), where CF is a β1 solvent and oDCB and Ani are β2 solvents, films prepared from CF exhibited substantially higher conductivity (σ_ave_ = 508 ± 107 S/cm, σ_max_ = 705 S/cm) compared to oDCB (σ_ave_ = 67.2 ± 49.8 S/cm, σ_max_ = 135 S/cm) and Ani (σ_ave_ = 0.57 ± 0.37 S/cm, σ_max_ = 1.7 S/cm) (Figure 6c). Similarly, for g_3_(2T‐TT), the β1 solvent (CF) again produced higher conductivity (σ_ave_ = 398 ± 98 S/cm, σ_max_ = 554 S/cm) than the β2 solvents oDCB (σ_ave_ = 137 ± 82 S/cm, σ_max_ = 259 S/cm) and Ani (σ_ave_ = 120 ± 63 S/cm, σ_max_ = 210 S/cm) (Figure 6d).

Notably, g_3_(2T‐T) provided an important control case, as all three solvents act as β1 solvents for this polymer (Figure 6e). In this case, the resulting films exhibited comparable levels of conductivity across solvents, confirming that the observed trends are governed by aggregation type rather than the choice of solvent itself. Specifically, the measured conductivities were σ_ave_ = 108 ± 28 S/cm (σ_max_ = 140 S/cm) for CF, σ_ave_ = 101 ± 25 S/cm (σ_max_ = 142 S/cm) for oDCB, and σ_ave_ = 101 ± 35 S/cm (σ_max_ = 154 S/cm). All conductivity data spanning a wide range of film thicknesses are summarized in Figure 6f–h. These results confirm that the observed conductivity variations are not due to differences in film thickness, as β1‐derived films consistently exhibited higher conductivities than β2‐derived films across the entire thickness range.

UV–vis–NIR spectroscopy of F4TCNQ‐doped films further revealed that the higher conductivity of β1‐derived films is accompanied by enhanced doping efficiency and increased formation of delocalized charged species (Figure S25a). Specifically, films with comparable thicknesses were prepared and doped using the same F4TCNQ doping protocol, after which their UV–vis–NIR absorption spectra were compared. β1‐derived films exhibited substantially stronger infrared polaronic absorption than β2‐derived films, indicating more effective charge transfer and higher charge‐carrier generation. Spectral deconvolution based on recently reported assignments of polaronic and bipolaronic absorption features [40, 42] further revealed a higher bipolaronic contribution in β1‐derived films compared to β2‐derived films (Figure S25b–d).

Mechanistically, this difference is likely associated with the distinct supramolecular packing structures of the films. Higher crystallinity and long‐range order in β1‐derived films can reduce energetic disorder and promote charge delocalization along the conjugated backbone, thereby enhancing both effective doping and charge transport. This interpretation is consistent with previous studies showing that increased crystallinity in F4TCNQ‐doped conjugated polymer films enhances conductivity primarily through improved carrier mobility and increased polaron delocalization [43]. Possible chirality‐related effects, including CISS, may also contribute by promoting oxidation over reduction during the doping process [7]. We will seek to validate this effect in future work.

## 3 Conclusion

In summary, we have established that the assembly pathway of OEG‐functionalized redox‐activate conjugated polymers is primarily governed by the aggregate type in solution, which is determined by solvent‐dependent solubility. We identified two distinct aggregate types: β1 aggregates, characterized by high torsional freedom and weak interchain coupling, and β2 aggregates, defined by planar backbones with stronger interchain coupling. The β1 and β2 aggregates can interconvert reversibly upon heating. While β2 aggregates lead to achiral agglomeration, β1 aggregates enable the emergence of chiral twist‐bend nematic mesophases. Our finding reveals a critical design rule that achieving a chiral LLC assembly pathway requires selecting solvents or processing temperatures that suppress β2 aggregation in favor of β1 aggregation. This LLC pathway not only facilitates supramolecular chirality but also drives superior long‐range order, resulting in films with significantly higher crystallinity and a multifold increase in electrical conductivity upon chemical doping across multiple polymer systems tested. This work provides a generalizable framework for modulating the morphology and electronic properties of redox‐active polymers through rational solvent selection and thermodynamic control of solution‐state aggregations.

## 4 Materials and Methods

All conjugated polymers were synthesized according to previously described procedures; p(g_4_2T‐TT) [39], p(g_3_2T‐TT) [38], p(g_3_2T‐T) [40], PBTTT‐EG3 [13], N2200‐OEG [44], PTQ‐4bO2 [45], and PTQ‐6bO [46]. The molecular number (Mn), weight‐average molecular weight (Mw), and polydispersity index (PDI) of all polymers are summarized in Table 1.

**TABLE 1:** Summary of polymers: molecular numbers, molecular weights, and PDI.

Polymers	*M*_n_ (g/mol)	*M*_w_ (g/mol)	PDI
p(g_4_2T‐TT)	28k	41k	1.5
p(g_3_2T‐TT)	16k	24k	1.5
p(g_3_2T‐T)	33k	49k	1.5
PBTTT‐EG3	34k	75k	2.2
N2200‐OEG	42k	92k	2.2
PTQ‐4bO2	110k	257k	2.3
PTQ‐6bO	48k	91k	1.9

### 4.1 Solution‐State Structure Characterization

All solutions were initially prepared in each solvent at concentrations of 1 mg/mL (for solubility tests) and 10 mg/mL (for solution state analysis). For the solvent screening study (1 mg/mL), dissolution occurred at room temperature in CF, at 80°C in CB, oDCB, CN, and Ani, and at 70°C in 3TCE, corresponding to 0.87, 0.78, 0.66, 0.83, and 0.95 of the respective solvent boiling points in Kelvin (T/T_b_). All solvents were anhydrous and purchased from Sigma Aldrich. Solution‐state morphology characterization was carried out using a CPOM (Nikon Eclipse Ci‐POL). Polymer solutions were sandwiched between two glass coverslips placed on a silicon wafer to enable reflection‐mode imaging. High‐concentration solutions were prepared by a drop‐and‐dry method, in which a defined volume of the original solution was evaporated, and the residue was redissolved in 5 µL of the same solvent. The resulting concentrated solution was homogenized by shearing between cover glasses. Prior to CPOM imaging, samples were subjected to thermal annealing cycles (10°C/min heating and cooling) in a Linkam stage to erase shear history and ensure equilibration at room temperature for 10 min. For transmission bright‐field optical measurements, one polarizer from the microscopy was removed.

UV–vis absorption spectra were recorded on an Agilent Cary‐60 spectrophotometer. For high‐temperature measurements, UV–vis spectroscopy was coupled to a custom‐built temperature control setup equipped with a TC300 heater temperature controller (Thorlabs). To ensure measurements were performed at equilibrium, the temperature was first raised close to the solvent boiling point, held for 10 min, and then decreased at a controlled cooling rate of 1°C/min. After reaching the target temperature, samples were equilibrated for an additional 30 min prior to measurement. All UV–vis spectra were fitted using Gaussian line shapes. This choice is physically motivated by the inhomogeneous broadening commonly observed in conjugated polymer systems, where variations in local conformation, intermolecular packing, and electronic coupling lead to a distribution of transition energies. In such systems, Gaussian functions provide an appropriate description of vibronic absorption features, in contrast to Lorentzian line shapes that correspond to homogeneous broadening mechanisms. This approach is widely established for disordered π‐conjugated systems and vibronic transitions [24]. For the fitting protocol, we first used the CF spectrum, where β1 aggregation is dominant and no β2‐specific low‐energy component is observed, to define the β1 vibronic features. The β1 component was described by two Gaussian peaks corresponding to the β1 (0–0) and β1 (0–1) transitions. The energy spacing between these two vibronic peaks was constrained to the range of approximately 1300–1400 cm^−^^1^, consistent with the well‐known coupling of π–π* transitions to thiophene ring‐breathing and stretching modes in polythiophene and related conjugated polymer systems [23]. For β2‐type solvents such as Ani, the β1 vibronic component was constrained using the β1 peak positions and vibronic spacing extracted from CF, allowing only a narrow variation of approximately ±5 nm to account for minor solvent‐dependent shifts. Under these constraints, an additional low‐energy component was then allowed to appear freely during fitting. This additional component consistently emerged at ∼650–660 nm and was assigned to β2 aggregation.

CD spectra of lyotropic solutions were measured with a JASCO‐1500 spectropolarimeter. For high‐concentration samples, drop‐and‐dry specimens prepared for CPOM analysis were used. To eliminate artifacts from linear dichroism and birefringence, a four‐scan averaging protocol was employed, involving 90° in‐plane and 180° out‐of‐plane sample rotations [26]. High‐temperature CD measurements were conducted following the same procedure as for UV–vis spectroscopy. For samples with absorption exceeding the detection limit, the bandwidth was increased to 10 nm to enhance the intensity of the incident light. The CD g‐factor quantifies the relative differential absorption of left‐ and right‐circularly polarized light with respect to the total absorption of unpolarized light [27]. It provides a normalized measure of circular dichroism independent of sample concentration, optical path length, or film thickness. The g‐factor is defined as [47, 48]:

$$g-\mathrm{facto}r_{\mathrm{abs}}=\frac{\Delta A}{A}=\frac{\theta }{32\;980\;\times \;A}=\frac{2\left(A_{L}-A_{R}\right)}{A_{L}+A_{R}}$$

where Δ*A* =  *A*_L_ − *A*_R_ is the difference in absorption between left‐ and right‐circularly polarized light, $\;A=\frac{A_{L}+A_{R}}{2}$ is the absorption of unpolarized light, θ is the ellipticity in millidegrees, and 32 980 is the conversion factor between ellipticity and absorbance units.

Solution SAXS experiments were carried out at the 16‐ID beamline of the National Synchrotron Light Source II at Brookhaven National Laboratory using a 13.5 keV beam and a Pilatus detector. To probe solution structures at elevated temperatures, additional SAXS measurements were performed at the Advanced Photon Source (beamline 12‐ID‐B, Argonne National Laboratory, Lemont, IL) using a 13.3 keV beam and a Pilatus detector with temperature capillary holder. To avoid beam‐induced damage, each sample was measured through 10–20 repeated scans with short exposure times of 1 s, taken at different positions across the capillary. The choice of capillary thickness was guided by x‐ray attenuation length: 0.4 mm capillaries were used for CF solutions, while 1.0 mm capillaries were used for Ani solutions. The resulting scattering profiles were then fit using the SASView program.

To directly visualize aggregates in solution, freeze‐drying experiments were performed followed by SEM (Hitachi‐4800) and AFM (Asylum Cypher) imaging. Polymer solutions were rapidly vitrified in a liquid propane–ethane mixture (63% propane and 37% ethane) and stored in liquid nitrogen, minimizing aggregation during cooling due to the high heat capacity of the cryogen. Samples were then transferred to Linkam stage at −130°C under vacuum, remaining below the melting points of the solvents used (−63.5°C for CF, −37°C for Ani). Complete solvent sublimation was confirmed after 8–10 h of monitoring under CPOM.

### 4.2 Film Printing and Chemical Doping

Thin films were deposited onto substrates using a meniscus‐guided blade coating technique. An OTS‐treated Si blade was positioned at a 3° printing angle with a 20 µm gap between the blade and substrate. The blade was translated linearly across the stationary substrate, maintaining the ink solution within the confined meniscus. Printing was initiated with a low solution concentration (20 mg/mL); as the solvent evaporated, the local concentration increased, promoting the in situ formation of mesophases typically observed only at higher equilibrium concentrations. To preserve these transient structures in the final film, coating was performed at low speeds within the evaporation regime, enabling near‐equilibrium assembly. Films were coated at 0.03–0.5 mm/s at 25°C for CF, 0.005–0.02 mm/s at 25°C for Ani, and 0.001–0.003 mm/s at 25°C for oDCB. The coating speed was adjusted depending on the targeted film thickness.

For chemical doping of thiophene‐based conjugated polymer with F4TCNQ, we employed a incremental concentration doping method to maximize film conductivity. Five doping solutions were prepared in acetonitrile with concentrations of 0.1, 0.033, 0.01, 0.0033, and 0.001 mg/mL. The solutions were applied sequentially to the film by pipetting, starting from the lowest concentration and progressing to the highest. At each step, the solution was allowed to remain on the film for 1 min before being removed with a gentle nitrogen blow. Following the final doping step, the samples were dried under vacuum for 10 min to remove residual solvent.

For chemical doping trials of quinoxaline‐ and naphthalene diimide‐based conjugated polymers, we tested two dopants, F4TCNQ and magic blue (tris(4‐bromophenyl)ammoniumyl hexachloroantimonate), dissolved in acetonitrile. The doping procedure followed the same method previously described. Neither polymer showed measurable conductivity (below the four‐point probe detection limit of 10 MΩ/sq), even when using dopant solutions with concentrations exceeding 1 mg/mL.

### 4.3 Film Morphology and Electrical Property Characterization

CPOM, CD, and AFM measurements of printed films were performed using the same instruments as those used for solution‐state analysis, following the procedures described above. Films for CPOM and AFM were prepared on silicon substrates, while CD measurements were conducted on glass substrates. Film conductivity was measured using a four‐point probe system (Ossila). At each spot, ten measurements were recorded and averaged; this was repeated at five different spots per sample (50 measurements total), and the results were averaged to obtain conductivity.

GIXD measurements were performed at beamline 11‐BM (CMS) at the National Synchrotron Light Source II, Brookhaven National Laboratory, with an incident beam energy of 13.5 keV on a 2D detector (PILATUS 800k) at a 260‐mm sample‐to‐detector distance. Samples were scanned for 10 s in a vacuum chamber. The x‐ray incident angle was set to be above (0.14°) and below (0.06°) the critical angle (≈0.1°) of the organic layer to probe the molecular packing throughout the entire film and near the top surface layer (penetration depth, approximately 5 nm). To assess crystallinity, the rDoC was extracted from GIXD patterns. The azimuthal angle χ was defined as = 0° corresponds to out‐of‐plane scattering and χ = 90° corresponds to in‐plane scattering. The rDoC values for lamellar, and π–π features were determined by integrating the corresponding intensity distributions according to:

$$rDoC\propto \int_{0}^{\pi /2}\sin\left(\chi \right)I\left(\chi \right)d\chi$$

where I(*χ*) is the scattering intensity as a function of χ. The paracrystalline disorder parameter, *g*, was calculated according to:

$$g=\sqrt{\frac{\Delta q}{2\pi q_{0}}}$$

where Δ*q* represents the breadth of the diffraction peak and *q*_0_ denotes the corresponding peak center position [49]. All diffraction peak parameters were obtained through curve fitting performed in OriginPro.

## Author Contributions

The manuscript was prepared through the contributions of all authors. All synthetic procedures and chemical characterization were performed by J. N. under the guidance of Prof. W. Y. Optical property measurements were conducted by S. J., N. A., and A. S., with guidance from Prof. Y. D. Structural characterization was carried out by S. J. under the supervision of Prof. Y. D. GIXD measurements were performed by Y.‐C. C., and the analysis was conducted by S. J. with assistance from Y.‐C. C. and P. B., under the guidance of Prof. Y. D. Device fabrication and analysis were performed by S. J., J. B., and C. K. P., also under the guidance of Prof. Y. D. The manuscript was drafted by S. J. and revised with input from all authors. All authors have reviewed and approved the final version of the manuscript.

## Conflicts of Interest

The authors declare no conflicts of interest.

## Supporting information




**Supporting File**: adma74130‐sup‐0001‐SuppMat.docx.

## Data Availability

The data that support the findings of this study are available from the corresponding author upon reasonable request.
