# Supplementary material for: Solvent‐Guided Chiral Assemblies and Chemical Doping of OEG‐Functionalized Conjugated Polymers
Source: Adv Mater. 2026 Jul 18;38(45):e74130. doi: 10.1002/adma.74130 (PMC13471970; doi:10.1002/adma.74130)
Supplement: Supplementary file 1 [file ADMA-38-e74130-s001.docx]

**Supporting Information**

**Solvent-Guided Chiral Assemblies and Chemical Doping of OEG-Functionalized Conjugated Polymers**

*Sanghyun Jeon^1^, Justin Scott Neu^2^, Nahyun Ahn^3^, Ayse Senlik^3^, Yen-Chi Chen^3^, Pravini S. Fernando^3^, Priyotosh Bairagya^3^, Chetan Kumar Prasad^4^, Janice Mihyun Baek^1^, Wei You^2^, and Ying Diao*^3,5^*

^1^Department Materials Science and Engineering, University of Illinois at Urbana-Champaign, 1304 W Green St, Urbana, IL 61801, USA

^2^Department of Chemistry, University of North Carolina at Chapel Hill, Chapel Hill, NC 27599, USA

^3^Department of Chemical and Biomolecular Engineering, University of Illinois at Urbana-Champaign, 600 South Mathews Avenue, Urbana, Illinois 61801, USA

^4^Department of Chemistry, University of Illinois at Urbana-Champaign, 505 South Mathews Avenue, Urbana, Illinois 61801, USA

^5^Beckman Institute for Advanced Science and Technology, University of Illinois at Urbana-Champaign, 405 N. Mathews Ave. M/C 251, Urbana, Illinois 61801, USA

KEYWORDS. Conjugated polymer, Self-assembly, Liquid crystal, Chirality, Solvent engineering

*Correspondence to: [yingdiao@illinois.edu](mailto:yingdiao@illinois.edu)

**Figure S1.** Concentration-dependent aggregation analysis for evaluating solvent-dependent aggregation behavior of p(g_4_2T-TT). **(a)** Optical microscopy images of Ani solutions with increasing polymer concentration from 1 to 15 mg mL⁻¹. Visible micro-aggregation first appears at 15 mg mL⁻¹, which is defined as the critical concentration for phase separation (C_s_). **(b)** Summary of C_s_ measured across 11 different solvents. **(c)** Comparison between the C_s_ and extent of β2 aggregation extracted from UV–Vis deconvolution. Solvents with higher C_s_ values generally exhibit lower β2 aggregate populations, supporting the correlation between effective backbone solubility and β2 aggregation tendency.

We quantitatively assessed solvent-dependent aggregation behavior using a concentration-dependent aggregation analysis, which is more appropriate for the present system. Specifically, we systematically increased the polymer concentration in each solvent and identified the concentration at which micron-sized aggregation first appeared under the optical microscope. We define this threshold as the critical concentration for phase separation (C_s_), a quantity conceptually related to what is referred to as the critical saturation concentration. For example, in Ani, the polymer solution remained homogeneous up to 13 mg mL⁻¹, while clear micro-aggregation was observed at 15 mg mL⁻¹ under optical microscopy (**Figure S1a**). Using this approach, we re-evaluated the solvent series investigated in this work (**Figure S1b**). For highly poor solvents such as methanol (MeOH), dimethyl sulfoxide (DMSO), hexane (Hex), propylene glycol methyl ether acetate (PGMEA), and toluene (Tol), aggregation occurred below 1 mg mL⁻¹, consistent with their classification as bad solvents. In contrast, trichloroethane (3TCE), chlorobenzene (CB), o-dichlorobenzene (oDCB), anisole (Ani), and chloronaphthalene (CN) exhibited aggregation onsets at approximately 10, 13, 15, 15, and 20 mg mL⁻¹, respectively. Chloroform (CF) showed no noticeable aggregation up to 100 mg mL⁻¹, above which the system directly formed an LLC phase.

Importantly, the measured C_s_ values show strong agreement with the independently determined β2 aggregation fractions obtained from UV–Vis deconvolution (**Figure S1c**). Solvents with higher C_s_ values, corresponding to better effective solubility, consistently exhibit lower β2 aggregate populations, whereas solvents with lower C_s_ values show stronger β2 aggregation tendencies, supporting our proposed framework that β2 aggregation is promoted under poorer backbone solvation conditions, which will be discussed in detail in later sections.

**Figure S2**. Bright field images of g4(2T-TT) solutions (1 mg/mL) in PGMEA, toluene, and DMSO, showing large undissolved microparticles due to poor solubility. In hexane and methanol, the polymer did not dissolve at all.

**Figure S3**. UV–Vis spectral deconvolution of p(g_4_2T-TT), p(g_3_2T-TT), and p(g_3_2T-T) solutions in various solvents. All solutions were prepared at 5 mg/mL and subjected to heating–cooling cycles (1 °C/min) to ensure equilibrium. Deconvolution separates contributions from the α phase (dispersed chains), vibronically correlated β1 aggregates, and the red-shifted β2 aggregation feature, highlighting the distinct solvent-dependent aggregation pathways. The β1 solvent is colored green, and the β2 solvent is colored orange.

**Figure S4.** UV–Vis spectral deconvolution of PBTTT-EG3, PTQ-6bO, PTQ-4bO2, and N2200-OEG solutions in various solvents. All solutions were prepared at 5 mg/mL and subjected to heating–cooling cycles (1 °C/min) to ensure equilibrium. Deconvolution separates contributions from the α phase (dispersed chains), vibronically correlated β1 aggregates, and the red-shifted β2 aggregation feature, highlighting the distinct solvent-dependent aggregation pathways. The β1 solvent is colored green, and the β2 solvent is colored orange.

**Figure S5.** Comparison of UV–Vis spectral fitting of the anisole solution using the α + β1 model without a β2 component. The experimental spectrum was fitted under the same physically constrained fitting protocol used in the main analysis, including constrained β1 vibronic spacing and peak-position bounds. Even after optimization to minimize χ², the α + β1 model fails to reproduce the low-energy absorption shoulder in the 650–660 nm region, resulting in a clear systematic deviation. In contrast, inclusion of an additional β2 component accurately captures this low-energy feature while preserving physically consistent β1 vibronic parameters. This comparison demonstrates that the β2 component is required to describe the spectrum and is not introduced as a result of overfitting.

**Figure S6**. Time-dependent CPOM images of concentrated **(a)** CF solutions (>200 mg mL⁻¹) and **(b)** anisole solutions (>200 mg mL⁻¹ at 130 °C), collected from 5 min to 1 h after reaching the target temperature. Minimal changes in the LLC textures are observed over time in both systems, confirming that the assemblies rapidly reach equilibrium and that 30 min is sufficient to ensure equilibrium conditions.

**Figure S7.** **(a)** AFM images of freeze-dried, concentrated p(g_4_2T-TT) solutions in CF at 200 mg/mL. Red arrows indicate chiral bundles composed of helical fibers. Yellow lines denote line-cuts used to estimate the inter-fiber spacing, while red lines indicate line-cuts used to determine the helical pitch. Corresponding height profiles from the yellow and red line-cuts are shown in **(b)** and **(c)**, respectively. All line-cut analyses were performed using ImageJ. Half-pitch (*p_1/2_*) length = 35.2 ± 5.4 nm, and a lateral stacking distance = 33.2 ± 5.5 nm

**Figure S8. (a)** AFM images of freeze-dried, concentrated p(g_4_2T-TT) solutions in Ani at 200 mg/mL. Yellow lines denote line-cuts used to estimate the inter-fiber spacing. Corresponding height profiles from the yellow line-cuts are shown in **(b)**. All line-cut analyses were performed using ImageJ. Lateral stacking distance = 11.4 ± 1.9 nm.

**Figure S9.** Concentration-dependent UV–Vis spectra of p(g42T-TT) solutions in chloroform (CF, left) and anisole (Ani, right) from 10 to 200 mg mL⁻¹. The characteristic spectral features associated with β1 aggregation in CF and β2 aggregation in anisole are largely preserved across the entire concentration range, with no emergence of a new β2-specific red-shifted component or systematic change in the relative aggregate population. Instead, the spectra progressively broaden with only minor peak shifts, indicating that increasing concentration promotes higher-order mesophase development and broader local packing distributions without substantially altering the primary β1/β2 aggregate identity.

**Figure S10.** Identification of intrinsic and artifact contributions in thin-film CD measurements and corresponding g-factor analysis. The bottom panel shows the absorbance spectrum, the middle panel shows the CD signal, and the top panel shows the calculated dissymmetry factor (g-factor). The shaded blue region corresponds to the absorption-active spectral range, where the CD signal originates from intrinsic electronic transitions and excitonic coupling associated with chiral supramolecular ordering. In contrast, the red-shaded region corresponds to wavelengths with negligible absorbance, where weak non-zero CD signals can produce artificially large g-factor values due to the small absorbance denominator and may arise from anisotropy- or scattering-induced artifacts. Therefore, all reported g-factor values in this work are extracted from the absorption band, specifically at the wavelength corresponding to the maximum CD signal within the absorption-active region, to ensure that they reflect intrinsic chiroptical properties rather than measurement artifacts. To avoid overestimation and ensure a physically meaningful comparison, we have revised our analysis of the g-factor. Instead of reporting the maximum g-factor across the entire spectral range, we now define the representative g-factor as the value calculated at the wavelength corresponding to the maximum CD signal within the absorption band. This approach restricts the analysis to absorption-active regions where the CD signal originates from intrinsic electronic transitions, rather than scattering artifacts.

**Figure S11.** Reversibility test of the temperature-mediated β2 ↔ β1 transition in concentrated anisole solutions (>200 mg mL⁻¹). **(a)** Temperature-dependent CPOM images collected during cooling from 130 to 50 °C, showing the disappearance of birefringent LLC textures below ~90 °C. **(b)** CD and UV–Vis spectra measured during heating from 25 to 130 °C, showing the emergence of the bisignate CD signal and the β2 → β1 transition. **(c)** CD and UV–Vis spectra measured during cooling from 130 to 50 °C, showing the disappearance of the bisignate CD signal and reversible recovery of the β2-associated absorption features. These cooling-cycle CPOM, CD, and UV–Vis measurements confirm that the temperature-mediated β2 ↔ β1 transition is reversible under the investigated conditions.

**Figure S12.** Temperature-dependent CPOM images of concentrated CF solutions (>200 mg mL⁻¹) collected during cooling from 20 to −20 °C. A significant reduction in birefringent LLC textures is observed below approximately −10 °C, indicating a transition away from the β1-associated ordered state toward a more strongly aggregated state. This behavior supports that the β1 ↔ β2 aggregation transition is governed by temperature-dependent backbone solubility.

**Figure S13. (a)** Solution WAXS analysis of g_4_2T-TT in chloroform (CF) and anisole (Ani). In CF, where the polymer forms the β1 state that supports chiral LLC assembly, no clear ordered packing feature is observed in the measured q range. In contrast, the Ani solution, which forms the β2 state that suppresses chiral LLC assembly, exhibits a pronounced scattering peak at q = 1.53 Å⁻¹, assigned to backbone π–π packing. Notably, no distinct OEG side-chain-related packing peak is observed in either CF or Ani. **(b)** Miscibility test using poly(ethylene glycol) dimethyl ether (Mn = 250, Sigma). This molecule, which closely resembles the OEG side chain, exhibited a high degree of miscibility with the organic solvents used in this study (up to ~50 vol%). Notably, it was also miscible with PGMEA, a solvent that was classified as insoluble in the initial screening.

**Figure S14.** Control experiments using non-OEG-substituted conjugated polymers. PBTTT-C14 and P3HT were tested across the same 11 solvents: chloroform, anisole, *o*-dichlorobenzene, chlorobenzene, dimethylformamide, 1-chloronaphthalene, propylene glycol methyl ether acetate, 1,4-dioxane, trichloroethylene, toluene, and ethanol. In all cases, no chiral LLC assembly was observed. CPOM images (left) show no birefringence, indicating the absence of liquid-crystalline ordering, while bright-field optical microscopy images (right) reveal only featureless, sheet-like morphologies at high concentrations. These results support that OEG side chains are critical for enabling the chiral LLC assembly pathway in the systems studied here.

**Table S1.** Model fitting parameters for 5 mg/mL solutions of p(g_4_2T-TT) in CF (left) and Ani (right). The *L_1_* value was fixed during fitting due to limitations of the low-q window. Because persistence length (*L_p1_*) estimation is strongly dependent on *L_1_*, no quantitative conclusions regarding Lp1 could be drawn in this case. The *L_2_* parameter was fixed at 80 nm, corresponding to the estimated contour length of an individual polymer chain.

| **Parameters** | **Value (CF)** | **Value (Ani)** |
| --- | --- | --- |
| *L_1_* (nm) | 200* | 200* |
| *L_p1_* (nm) | >>*R_1_* | >>*R_1_* |
| *R_1_* (nm) | 8.64 ± 0.23 | 8.40 ± 0.64 |
| *q_1_* (Å^-1^) | - | 0.043 ± 0.001 |
| *FWHM_1_* | - | 0.0182 ± 0.0002 |
| *w_1_* | - | 0.675 ± 0.05 |
| *L_2_* (nm) | 80* | 80* |
| *L_p2_* (nm) | 7.02 ± 2.5 | 41.6 ± 2.2 |
| *R_2_* (nm) | 1.22 ± 0.08 | 1.39 ± 0.005 |
| *q_2_* (Å^-1^) | 0.185 ± 0.02 | 0.2213 ± 0.0001 |
| *FWHM_2_* | 0.05 ± 0.01 | 0.1134 ± 0.003 |
| *w_2_* | 0.27 ± 0.01 | 0.865 ± 0.06 |
| *PDI* | 0.2 | 0.2 |

*Fixed during the fitting.

**Table S2.** Description of the fitting parameters:

| **Parameters** | **Description** |
| --- | --- |
| *L_1_* | Length of flexible cylinder 1, corresponding to the length of fiber |
| *L_p1_* | Persistence length of flexible cylinder 1, corresponding to the persistence length of fiber |
| *R_1_* | Radius of flexible cylinder 1, corresponding to the radius of fiber |
| *q_1_* | Location of the pseudo-Voigt peak |
| *FWHM_1_* | Full width half maximum of the pseudo-Voigt peak |
| *w_1_* | Lorentzian fraction of the pseudo-Voigt peak |
| *q_2_* | Location of the Lorentz peak |
| *FWHM_2_* | Full width half maximum of the Lorentz peak |
| *L_2_* | Length of flexible cylinder 2, corresponding to the length of individual polymer |
| *L_p2_* | Persistence length of flexible cylinder 2, corresponding to the persistence length of individual polymer |
| *R_2_* | Radius of flexible cylinder 2, corresponding to the radius of individual polymer |
| *q_2_* | Location of the pseudo-Voigt peak |
| *FWHM_2_* | Full width half maximum of the pseudo-Voigt peak |
| *w_2_* | Lorentzian fraction of the pseudo-Voigt peak |
| *PDI* | Polydispersity in the radius |

**Figure S15**. The SAXS profile of β2-type solutions (5 mg/mL, Ani) at elevated temperature (130 °C), a condition in which β2 aggregation disappears as confirmed by optical analysis (Figure 2), was well fitted with a two flexible cylinder model and a single weak pseudo-Voigt feature. This result indicates weak interchain coupling without higher order packing, closely resembling the characteristics of the β1 state. The table below summarizes the model fitting parameters.

| **Parameters** | **Value (Ani-130**°C**)** |
| --- | --- |
| *L_1_* (nm) | 200* |
| *L_p1_* (nm) | >>*R_1_* |
| *R_1_* (nm) | 9.40 ± 0.02 |
| *q_1_* (Å^-1^) | - |
| *FWHM_1_* | - |
| *w_1_* | - |
| *L_2_* (nm) | 80* |
| *L_p2_* (nm) | 7.9 ± 0.3 |
| *R_2_* (nm) | 1.023 ± 0.005 |
| *q_2_* (Å^-1^) | 0.20 ± 0.01 |
| *FWHM_2_* | 0.20 ± 0.05 |
| *w_2_* | 0.27 ± 0.01 |
| *PDI* | 0.2 |

*Fixed during the fitting.

**Table S3.** Model fitting parameters for 5 mg/mL solutions of p(g_4_2T-TT) in Ani at 130°C. The *L_1_* value was fixed during fitting due to limitations of the low-q window. Because persistence length (*L_p1_*) estimation is strongly dependent on *L_1_*, no quantitative conclusions regarding Lp1 could be drawn in this case. The *L_2_* parameter was fixed at 80 nm, corresponding to the estimated contour length of an individual polymer chain.

**Figure S16.** Reproducibility of β1-derived blade-coated films. CPOM images of four independently prepared β1-derived films fabricated from chloroform (CF) solutions under identical evaporation-regime blade-coating conditions. All samples show consistent film morphology, aligned fibrillar textures, and comparable birefringence over large areas, demonstrating that the β1-derived film structure is highly reproducible and not dominated by sample-to-sample variation from the coating process.

**Figure S17.** CD (g-factor) and absorption spectra of highly concentrated solutions (200 mg/mL) and the corresponding films fabricated by evaporation-regime blade coating from CF (β1 aggregation) and anisole (β2 aggregation) solutions. **(c)** Summary of g-factors for solutions (red bars) and printed films (blue bars) fabricated under the evaporation-regime blade-coating process for various polymers, solvents, and temperatures. On the x-axis, orange labels indicate β2-type aggregation conditions, while green labels indicate β1-type aggregation conditions. These results demonstrate that only β1-type solutions exhibit significant supramolecular chirality and, crucially, that this chirality can be faithfully transferred from solution into solid-state films.

**Figure S18.** AFM images of blade-coated p(g_4_2T-TT) film in CF. Yellow lines denote line-cuts used to estimate the inter-fiber spacing. Corresponding height profiles from the yellow line-cuts show inter fiber distance is 27.9 ± 10.4 nm. All line-cut analyses were performed using ImageJ.

**Figure S19.** **(a)** GIXD line cuts of the β1 film collected along azimuthal angles from 7° (out-of-plane) to 85° (in-plane). **(b)** Peak deconvolution of the out-of-plane line cut, showing lamellar packing (black), side-chain packing (turquoise), and π–π stacking (purple). **(c)** Peak deconvolution of the in-plane line cut, showing lamellar packing (black), polymer backbone (blue), side-chain packing (turquoise), and π–π stacking (purple). **(d)** GIXD line cuts of the β2 film collected along azimuthal angles from 7° (out-of-plane) to 85° (in-plane). **(e)** Peak deconvolution of the out-of-plane line cut, showing lamellar packing (black), side-chain packing (turquoise), and π–π stacking (purple). **(f)** Peak deconvolution of the in-plane line cut, showing polymer backbone (blue), and π–π stacking (purple). **(g)** 2D GIXD pattern of the β2 film, with the angular sector (55°–70°) and q-range (0–2 Å⁻¹) used for the line cut highlighted in red. Lin cut in the angle of 55°–70°, showing a π–π stacking peak at q = 1.65 Å^-1^. Quantitative spacing analysis is summarized in **Table S4**.

|  | **CF (β1)** | **Ani (β2)** |
| --- | --- | --- |
| Lamellar (nm) | 1.57 ± 0.04 | 1.53 ± 0.04 |
| Backbone (nm) | 1.34 ± 0.03 | 1.34 ± 0.06 |
| Sidechain (Å) | 4.69 ± 0.03 | 4.46 ± 0.04 |
| π-π (Å) | 3.90 ± 0.01 | 3.81 ± 0.01 |
| Helical π-π (Å)  rDoC_π-π_ | 3.93 ± 0.01  4.15 | -  1.48 |
| rDoC_lamellar_ | 0.87 | 0.21 |
| Paracrystallinity, ~g (%) | 10.8 | 13.2 |

**Table S4**. Summary of GIXD peak-fitting results for β1- (CF) and β2- (Ani) type films.

**Figure S20**. CD spectra (g-factor and absorption) of p(g_4_2T-TT) solutions under various solvent conditions. Solvents classified as β1 are indicated in green, while β2 solvent conditions are indicated in orange. All β1 solvent solutions were prepared at 200 mg/mL, whereas β2 solvent solutions were prepared at 100 mg/mL. All β1 solvents exhibit clear bisignate CD signals, while β2 solvents show no detectable CD response.

**Figure S21.** CD spectra (g-factor and absorption) of p(g_3_2T-TT), and p(g_3_2T-T) solutions under various solvent conditions. Solvents classified as β1 are indicated in green, while β2 solvent conditions are indicated in orange. All β1 solvent solutions were prepared at 200 mg/mL, whereas β2 solvent solutions were prepared at 100 mg/mL. All β1 solvents exhibit clear bisignate CD signals, while β2 solvents show no detectable CD response.

**Figure S22**. CD spectra (g-factor and absorption) of PBTTT-EG3, PTQ-6bO, and PTQ-4bO2 solutions under various solvent conditions. Solvents classified as β1 are indicated in green, while β2 solvent conditions are indicated in orange. All β1 solvent solutions were prepared at 200 mg/mL, whereas β2 solvent solutions were prepared at 100 mg/mL. All β1 solvents exhibit clear bisignate CD signals, while β2 solvents show no detectable CD response.

**Figure S23.** CD spectra (g-factor and absorption) of N2200-OEG solutions under various solvent conditions. Solvents classified as β1 are indicated in green, while β2 solvent conditions are indicated in orange. All β1 solvent solutions were prepared at 200 mg/mL, whereas β2 solvent solutions were prepared at 100 mg/mL. All β1 solvents exhibit clear bisignate CD signals, while β2 solvents show no detectable CD response.

**Figure S24.** CPOM images of LC phases are shown for various conjugated polymers under solution conditions corresponding to β1 solvents, which promote chiral LC formation. The images reveal birefringent textures characteristic of lyotropic liquid crystalline assemblies.

**Figure S25**. UV–Vis–NIR analysis of F4TCNQ-doped films prepared from different solvent conditions. **(a)** UV–Vis–NIR absorption spectra of doped films prepared from CF, Ani, and oDCB under identical doping conditions. Films were prepared with comparable thicknesses: CF, 121 ± 14 nm; anisole, 134 ± 16 nm; and oDCB, 127 ± 11 nm. Spectral deconvolution of doped films prepared from **(b)** CF, **(c)** Ani, and **(d)** oDCB into residual neutral polymer absorption, polaronic P1 and P2 bands, and bipolaronic BP contributions.
